# Depression, anxiety, insomnia, stress, and the way of coping emotions as risk factors for ischemic stroke and their influence on stroke severity: A case–control study in Lebanon

**DOI:** 10.3389/fpsyt.2023.1097873

**Published:** 2023-02-21

**Authors:** Elise Maalouf, Souheil Hallit, Pascale Salameh, Hassan Hosseini

**Affiliations:** ^1^Life and Health Sciences Department, Paris-Est University, Paris, France; ^2^School of Medicine and Medical Sciences, Holy Spirit University of Kaslik, Jounieh, Lebanon; ^3^Applied Science Research Center, Applied Science Private University, Amman, Jordan; ^4^Research Department, Psychiatric Hospital of the Cross, Jal Eddib, Lebanon; ^5^School of Medicine, Lebanese American University, Byblos, Lebanon; ^6^INSPECT-LB: Institut National de Santé Publique, Épidémiologie Clinique et Toxicolo-gie-Liban, Beirut, Lebanon; ^7^Medical School, University of Nicosia, Nicosia, Cyprus; ^8^Faculty of Pharmacy, Lebanese University, Hadat, Lebanon; ^9^Faculté de Santé, UPE-C, Université Paris-Est Créteil, Créteil, France; ^10^Hopital Henri Mondor, APHP, Créteil, France

**Keywords:** ischemic stroke, depression, anxiety, perceived stress, insomnia, cognitive reappraisal, expressive suppression

## Abstract

**Background:**

Stroke is a leading cause of disability and death worldwide. There are numerous debates regarding the relationship between depression, anxiety, insomnia, perceived stress, and ischemic stroke. Moreover, no research on the efficacy of emotion regulation, which is critical for various components of healthy affective and social adaptability, is being conducted. To the best of our knowledge, this is the first study in the MENA region to shed light on the relationship between these conditions and stroke risk, aiming to determine whether depression, anxiety, insomnia, stress, and the way of coping with emotions may be risk factors for ischemic stroke occurrence and to further investigate the ability of two specific types of emotion regulation (cognitive reappraisal and expressive suppression) as possible moderators of the relationship between these psychological diseases and ischemic stroke risk. As a secondary objective, we sought to determine how these pre-existing conditions affect stroke severity levels.

**Methods:**

This is a case–control survey study involving 113 Lebanese inpatients with a clinical diagnosis of ischemic stroke admitted in hospitals and rehabilitation centers in Beirut and Mount Lebanon, and 451 gender-matched volunteers without clinical signs of stroke as controls recruited from the same hospitals as the cases or attending outpatient clinics for illnesses or treatments unconnected to stroke or transient ischemic attack, as well as visitors or relatives of inpatients (April 2020–April 2021). Data was collected by filling out an anonymous paper-based questionnaire.

**Results:**

According to the outcomes of the regression model, depression (aOR: 1.232, 95%CI: 1.008–1.506), perceived stress (aOR: 1.690, 95%CI: 1.413–2.022), a lower educational level (aOR: 0.335, 95%CI: 0.011–10.579), and being married (aOR: 3.862, 95%CI: 1.509–9.888) were associated with an increased risk of ischemic stroke. The moderation analysis revealed that expressive suppression had a significant moderating effect on the relationship between depression, anxiety, perceived stress, insomnia, and ischemic stroke risk, resulting in an increased risk of stroke incidence. In contrast, cognitive reappraisal significantly reduced the risk of ischemic stroke by moderating the association between ischemic stroke risk and the following independent variables: perceived stress and insomnia. On the other hand, our multinomial regression model revealed that the odds of moderate to severe/severe stroke were significantly higher in people with pre-stroke depression (aOR: 1.088, 95% CI: 0.747–1.586) and perceived stress (aOR: 2.564, 95% CI: 1.604–4.100) compared to people who had never had a stroke.

**Conclusion:**

Despite several limitations, the findings of our study suggest that people who are depressed or stressed are more likely to have an ischemic stroke. Consequently, additional research into the causes and effects of depression and perceived stress may provide new directions for preventive strategies that can help reduce the risk of stroke. Since pre-stroke depression and perceived stress were also found to be strongly correlated with stroke severity, future studies should evaluate the association between pre-stroke depression, perceived stress, and stroke severity to gain a deeper understanding of the complex interaction between these variables. Lastly, the study shed new light on the role of emotion regulation in the relationship between depression, anxiety, perceived stress, insomnia, and ischemic stroke.

## Background

Stroke is one of the leading causes of disability and mortality worldwide (1, 2). Both non-modifiable (such as age, gender, race or ethnicity, and heredity) and modifiable (such as diabetes, atrial fibrillation, smoking, dyslipidemia, and hypertension) risk factors for stroke have been widely investigated (3). These factors can pave the way for a stroke, but little is understood about how emotions and feelings might influence a stroke.

Following a stroke, which happens when blood flow to a region of the brain is cut off or reduced, emotional alterations are typical (4, 5). Because the damage alters the brain, which determines behavior and reactions, stroke survivors frequently experience more negative emotional states than they did originally (4, 5). Indeed, the American Stroke Association associates post-stroke patients’ conditions with irritability, perplexity, anger, and depression (6). Moreover, evidence shows that negative emotions may contribute to an increase in stroke risk. Researchers discovered in a 2021 worldwide INTERSTROKE research published in the European Heart Journal that anger and other distressing feelings, such as sorrow, depression, or anxiety, were associated with a 30% increase in the risk of stroke 1 h after an episode (7).

Besides, depression and anxiety are common, debilitating mental health problems, according to the WHO (8). Indeed, the American Psychological Association (APA) defines major depression as a mood disorder characterized by persistent sadness and other symptoms of a major depressive episode but without accompanying episodes of mania or hypomania or mixed episodes of depressive and manic or hypomanic symptoms (9). Anxiety disorders, on the other hand, were defined by the APA as any of a group of disorders with the emotional state of fear, worry, or excessive apprehension as their central organizing theme (9). Insomnia, which is a common symptom in 30 to 90% of psychiatric disorders, might be found in these aforementioned mental conditions but could also be identified separately. It is described as persistent problems with sleep initiation, maintenance, consolidation, or quality despite enough sleep time and opportunity, leading to some type of daily impairment (10).

Moreover, while some individuals adjust to challenges and changes more effectively, others develop adjustment issues or struggle to manage their emotions, and this pressure can become stressful, expanding the risk of suffering a stroke. Additionally, feelings about the unpredictability and uncontrollability of one’s life, how frequently one needs to deal with disappointments, how much change happens in one’s life, and confidence in one’s capacity to cope with issues or obstacles can all presumably involve perceived stress (PS) (11, 12). The ability to manage one’s emotions is critical for human adaptability. People can sometimes manipulate or mentally alter the interpretation of emotionally negative stimuli. When combined, cognitive reappraisal (CR) may be the primary option for dealing with potential challenges. At other times, people suppress their feelings during social encounters. Thus, they conceal their emotional behavior, behave spontaneously, or reappraise rationally in a way that reduces their emotional response (13, 14).

Few studies have examined the relationship between mental health issues and ischemic stroke in the general population. While negative emotions and mental health were traditionally regarded as being independent from physical health, there is increasing evidence that the two are inextricably intertwined. New research connects anxiety, stress, and depression to an increase in stroke cases (15–17). Depression was shown to be related to a considerably higher risk of stroke morbidity and death in a systematic review and meta-analysis investigating the relationship between depression and the risk of developing stroke in adults (15). In light of this, prospective research indicated a two-fold greater risk of a first-ever stroke linked with past depression, even after adjusting for confounders (18). Furthermore, depression was linked to an increased risk of all strokes and ischemic stroke in a large multicenter (22 low- and middle-income countries) case control study (19). The association between anxiety and stroke, on the other hand, was less established, with fewer research and inconsistent results. A meta-analysis found substantial evidence for a link between anxiety and stroke, with the pooled risk of stroke increasing by 24% among individuals suffering from anxiety disorders (16). There is scant proof that the risk of stroke may be elevated within 3 years after the diagnosis of anxiety and that the risk of stroke may be enhanced further for people with severe anxiety (16). These findings contrasted with a previous study that reported no link between anxiety disorders and stroke; only anxiety symptoms were associated with stroke in the short term (20). As a result, these results require confirmation.

Nonetheless, sleep and stroke research has mostly emphasized on the links between sleep apnea (21–24), sleep-disordered breathing (24, 25), sleep length (24, 26), and stroke; insomnia has received far less attention. The few research that have investigated the links between insomnia and stroke have discovered that insomnia eventually leads to stroke (24, 27, 28), and that this link is especially prominent in young adults (29). Individuals who experienced multiple insomnia symptoms were 10% more likely to have an ischemic stroke than non-symptomatic adults (30). People who had chronic insomnia had a higher risk of stroke than people who had intermittent insomnia, and both groups had a higher risk than people whose insomnia ended during the study period, according to a large longitudinal experiment (29). Furthermore, diabetes, hypertension, and high cholesterol have all been related to an increased risk of stroke among insomniacs (29).

Besides, stress is an inescapable part of everyday life. Whilst PS is often recognized by the general population as a risk factor for stroke (30), scientific research on this topic is scarce. A few studies have revealed that significant perceived psychological stress, different stressful life events, and inability to discover effective coping methods in stressful conditions are all separately related with an elevated risk of stroke (31–34). However, conflicting findings have been reported. Research that looked at perceived psychological stress, psychological discomfort, or recent stressful life events found no link or conclusive evidence to support the relationship between these variables and a non-fatal or total stroke (3, 17, 35). Although the existence of a connection between PS and the risk of stroke is still unclear, stress can increase the risk of cerebrovascular disease by modulating sympathomimetic activity, affecting blood pressure reactivity, cerebral endothelial function, coagulation, or heart rhythm (36–38). In neurological practice and research, emotional stress appears to be an undervalued risk factor (36).

Furthermore, no research is being conducted on particular emotional pathways, such as the efficacy of emotion regulation (ER), which is crucial for diverse components of healthy affective and social adaptability (13, 39), in reducing the risk of stroke. The empirical work of ER stemmed from the stress and coping literature, precisely Lazurus and colleagues’ conceptualizations of coping strategies (40, 41). Following that, and with the insight that some regulation strategies may be beneficial while others are deleterious, Gross proposed a modeling approach to ER in 1998, emphasizing the timing of a regulation strategy as critical to its impact and repercussions (42). He distinguished between antecedent-focused regulation (intervention occurs early and is centered on changing the effect of emotion-generating cues) and response-focused regulation [engages late in the process and aims to modify emotional output (e.g., action, expression)] (42–44). Notably, Gross and colleagues focused on the factors that are associated with and have implications for expressive suppression (ES) and cognitive reappraisal (CR), two key ER strategies reflective of these intervention points (43, 44). CR, an antecedent-focused strategy, is described as the endeavor to reinterpret an emotion-eliciting circumstance in a way that affects its meaning and emotional significance (45, 46). The attempt to conceal, inhibit, or minimize ongoing emotion-expressive behavior was referred to as ES, a response-focused strategy (46, 47). CR, according to Gross’ model, enables the implementation and production of interpersonal behavior that is suitably focused on social interaction. Conversely, ES takes place late in the emotion-generation process. It modulates the behavioral aspect of emotional reactions while maintaining the perceptual and physiological experience of negative emotion (39, 46). Adopting CR to manage emotions was therefore connected with stronger affective, social functioning, and well-being patterns than ES (13), which was linked to psychopathology, social dysfunction, and depression (48). Moreover, emerging scientific research suggests that suppressing emotions might contribute to increased cardiovascular problems, particularly in women (49). However, it is yet to be determined if the choice of ER strategy influences the likelihood of stroke or could potentially modulate the impact of other diseases, particularly psychological diseases, that were ostensibly influenced by ER strategies, on stroke risk.

Aside from the previously held belief that the coexistence of mental and cerebrovascular disease is simply due to their high prevalence, shared risk factors or pathomechanisms, and complex causal relationships, it has been demonstrated that pre-existing psychological disorder predicts more severe strokes on admission and negatively affects functional and cognitive outcomes after stroke (50, 51). Despite the current evidence, there is still much to be confirmed about the effects of different psychological disorders and ER strategies on stroke severity. Specific conditions, such as depression, anxiety, insomnia, and PS, have been studied primarily with a focus on the assessment of stroke risk (52). In the Middle East, particularly in Lebanon, studies examining whether the aforementioned triggers cause a stroke or their impact on the severity of a stroke at admission are extremely rare. In fact, a dearth of data was noticed.

To the best of our knowledge, this is the first study in the MENA region to shed light on the relationship between these conditions and stroke risk, aiming to determine whether depression, anxiety, insomnia, stress, and the way of coping with emotions may be risk factors for ischemic stroke occurrence and to further investigate the ability of two specific types of ER as possible moderators of the relationship between these psychological diseases and ischemic stroke risk. As a secondary objective, we sought to determine how these pre-existing conditions affect stroke severity levels.

## Methods

### Study design

**Figure 1:**
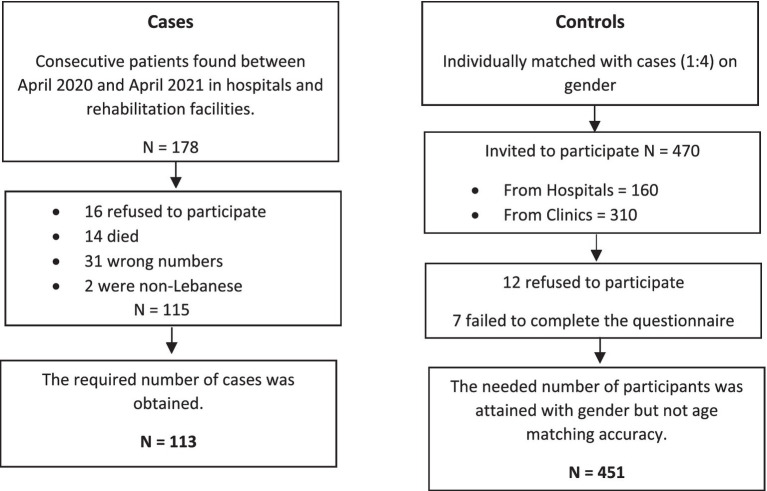
Participants’ flowchart showing cases of ischemic stroke and matched controls.

A case–control survey was carried out. The participants were given an informed consent that outlined the study’s objectives, benefits, concerns, and the confidential nature of the information gathered. The participation was entirely optional, with no financial incentive. The information was acquired between April 2020 and April 2021 by filling out an anonymous paper-based questionnaire, completed in-person. The same methodology has been used in a previous paper (53). Details of this method are presented in Figure 1.

Cases were Lebanese people aged 18 and above who survived an ischemic stroke admitted, during the observation period or previously (no more than 1 month prior to the observation period), in seven hospitals and rehabilitation centers in Beirut and Mount Lebanon were included: they should have underwent computed tomography (CT) and/or magnetic resonance imaging (MRI) to establish the diagnosis of ischemic stroke. Radiologic diagnosis had to be clinically confirmed by the physician diagnosis in the patient’s file. An ischemic stroke was defined as an acute infarction with no indications of bleeding on imaging (54). Exclusion criteria were the absence of written approval, the absence of clinical information or a CT/MRI record, and the presence of hemorrhagic stroke.

As for controls, we identified four volunteers for each case, gender-matched, with no clinical indications of stroke (confirmed by CT scan) or a history of stroke were included during the study period. The controls were recruited from the same hospitals as the patients or from the general population. Individuals hospitalized or attending outpatient clinics for illnesses or treatments unconnected to stroke or transient ischemic attack, as well as visitors or relatives of inpatients, were among the hospital-based sources. Controls were excluded if they were unable to provide written consent for the survey to be performed.

### Minimal sample size calculation

By using Epi info software, a sample of 564 participants (113 Lebanese patients with ischemic stroke and 451 controls) was constructed with a margin of error of 5%, a power of 80%, and an allocation number of 1:4 to allow for accurate bivariate and multivariable analysis. It was determined based on a stated estimate of 17% of controls having mental health concerns (55), and the OR of stroke within 30 days of a hospital admission for a mental health concern was reported to be 3.11 (56); we used an OR = 2 to have a more conservative sample size.

### Questionnaire and variables

The questionnaire was written in Arabic, the native language of Lebanon, and needed around 60 min to answer. Participants’ demographic characteristics were assessed, such as age, gender, and marital status were surveyed, as well as social factors such as region, level of education, occupation, and monthly income; in addition, health issues like personal history of hypertension, hyperlipidemia, diabetes, cardiovascular disease, atrial fibrillation, cancer, stroke type, date of stroke and severity by using the NIHSS score measuring multiple aspects of brain function, including consciousness, vision, sensation, movement, speech, and language, and ranges from 0 to 42. The levels of stroke severity as measured by the NIHSS scoring system were categorized as follows: no stroke symptoms: 0; minor stroke: 1–4; moderate stroke: 5–15; moderate to severe stroke: 16–20; and severe stroke: 21–42 (57).

Depression, anxiety, stress, insomnia, and the emotional coping method used prior to the ischemic stroke or enrollment at the time of stroke hospitalization were assessed by the questionnaire for all cases; for patients unable to communicate adequately, surrogate respondents who were considerate of their psychological states (a guardian or parent who was constantly present with the case) answered to the below scales.

- Hamilton depression rating scale (HDRS)

The HDRS, which was validated in Lebanon, is the most extensively used tool for evaluating depressive symptoms (58). Despite having 21 items, the scoring is solely based on the first 17. Eight of these 17 are evaluated on a 5-point scale ranging from 0 (not present) to 4 (severe) (59), and nine are scored from 0 to 2. The overall score is determined by summing the answers to the 17 questions. As a result, the total HDRS score varied from 0 to 52 points. Higher scores reflect a higher level of depression (59) (α Cronbach = 0.905).

- Hamilton anxiety rating scale (HAMA)

The HAMA, which was previously validated in Lebanon, is used to assess the severity of anxiety symptoms (60). The scale consists of 14 items, each described by a set of symptoms, and evaluates both psychic anxiety (mental agitation and psychological distress) and somatic anxiety (physical complaints related to anxiety). Each item is scored on a scale of 0 (not present) to 4 (severe), with a total score range of 0–56 (61). Higher scores imply higher levels of anxiety (α Cronbach = 0.938).

- Perceived stress scale (PSS)

The PSS includes 10 items that evaluate individual perceived stress levels during the prior month. Examples of items: “In the last month, how often have you been upset because of something that happened unexpectedly?,” “In the last month, how often have you felt that you were unable to control the important things in your life?,” “In the last month, how often have you felt nervous and stressed?.” The answers are scored on a range of 0 (never) to 4 (very often) (62). The overall score can vary between 0 to 40, with higher values indicating greater perceived stress (α Cronbach = 0.980).

- Lebanese insomnia scale (LIS-18)

The LIS-18 is an 18 items questionnaire generated in Lebanon to measure the extent of insomnia in Lebanese adult patients (63). Higher scores reflect higher insomnia (α Cronbach = 0.963).

- Emotion regulation questionnaire (ERQ)

A 10-item scale designed to measure respondents’ tendency to regulate their emotions in two ways: (1) Cognitive Reappraisal (6 items, possible range, 6–42, e.g., “I control my emotions by changing the way I think about the situation I’m in”) and (2) Expressive Suppression (4 items, possible range, 4–28, e.g., “I control my emotions by not expressing them”) (64). Respondents answered each item on a 7-point Likert-type scale ranging from 1 (strongly disagree) to 7 (strongly agree). Higher scores reflect more CR and more ES, respectively, (Cronbach’s alpha for CR = 0.988 and ES = 0.981). This scale has recently been validated in Lebanon (65).

### Statistical analysis

Data were analyzed using SPSS software version 23. A descriptive analysis was carried out using frequencies and percentages for categorical variables and means and standard deviations for continuous measurements. A calculation of the skewness and kurtosis revealed that the distribution was normal across all scales; values for asymmetry and kurtosis between −1 and + 1 were regarded acceptable in order to corroborate the normal univariate distribution (66). Afterwards, a bivariate analysis was performed to identify potential risk factors for ischemic stroke. Since all scales had a normal distribution, the Student’s test was utilized to compare means between two groups. The Chi-square and Fisher exact tests were used to compare percentages between two groups. A value of p less than 0.05 was considered significant.

A backward stepwise regression model was performed to investigate the odds ratio (OR) with a 95% CI of depression, anxiety, PS, insomnia, CR and ES among participants with ischemic stroke and the control group. Omnibus test was supposed to be significant to indicate that at least one of the introduced covariates had a substantial effect on the dependent variable. Hosmer-Lemeshow test was supposed to be non-significant in order to demonstrate the test’s adequacy. All covariates with a value of p of less than 0.2 in the bivariate analysis were included in the logistic regression models. The CI was set at 95%, and a value of p less than 0.05 was considered significant.

A backward stepwise moderation analysis was conducted to determine whether the choice of ER strategy may mitigate the impact of the mental disorder and hence influence the occurrence of ischemic stroke. First and foremost, we created a new variable by combining the putative moderators (CR and ES) with the corresponding independent variable. For instance, CR*HDRS, ES*HDRS, and so on. Second, we examined their significance and, as a result, the feasibility of integrating the new variable in each model alongside the corresponding direct impact of the respective independent variable and the two moderators. Consequently, four distinct models were developed. Following that, we incorporated the new variables, as well as all of the direct impact connected to the independent variable and the two moderators and evaluated the model as a whole. Omnibus test was confirmed to be significant to indicate that at least one of the introduced covariates had a substantial effect on the dependent variable. Hosmer-Lemeshow test was confirmed to be non-significant in order to demonstrate the model adequacy to data. The CI was set at 95%, and a value of *p* less than 0.05 was considered significant.

On the other hand, a multinomial logistic regression was conducted, taking the levels of stroke severity, as measured by the NIHSS scoring system, as the dependent variable. The Chi-square test was used to compare categorical variables, while the ANOVA test was employed to compare three means. All variables with a *p* < 0.2 were included in the final model as independent variables. The significance level was set at a *p* < 0.05.

## Results

### Effect of variables on ischemic stroke risk

#### Demographic data of patients with ischemic stroke and the control group (N = 564)

The demographic factors are summarized in Table 1 (previously depicted in a prior study (53)). When compared to non-ischemic stroke participants, ischemic stroke patients had a substantially higher mean age (65.5 vs. 62.9) and a higher percentage of married individuals (75.2% vs. 63.4%). Furthermore, according to the results of our study, ischemic stroke patients had a considerably lower educational level but a higher monthly income than controls. Further, preexisting physical comorbidities, such as hypertension (72.6% vs. 56.3%, *p* = 0.002), dyslipidemia (57.5% vs. 45.5%, *p* = 0.027), diabetes (36.3% vs. 26.6%, *p* = 0.048), heart diseases (42.5% vs. 11.8%, *p* < 0.001), atrial fibrillation (30.1% vs. 8.0%, *p* < 0.001) and asthma-COPD (38.9% vs. 26.2%, *p* = 0.008) were also more common in patients with ischemic stroke compared to those who did not.

**Table 1:** Bivariate analysis of demographic factors associated with ischemic stroke.

Variable	Presence of ischemic stroke (N = 113)	Absence of ischemic stroke (N = 451)	Value of *p*
	Mean ± SD		**0.035**
Age	65.5 ± 11.9	62.9 ± 11.6	
Gender	N (%)		1
Male	51 (45.1%)	203 (45.0%)	
Female	62 (54.9%)	248 (55.0%)	
Marital status			**0.020**
Single	13 (11.5%)	135 (29.9%)	
Married	85 (75.2%)	286 (63.4%)	
Divorced	3 (2.7%)	20 (4.4%)	
Widowed	12 (10.6%)	10 (2.2%)	
Educational level			**<0.001**
Primary-Complementary	61 (54.0%)	141 (31.3%)	
Secondary	20 (17.7%)	164 (36.4%)	
University	32 (28.3%)	146 (32.4%)	
Monthly income			**<0.001**
Low (<1,000 USD)	66 (58.4%)	290 (64.3%)	
Intermediate (1000–2000 USD)	25 (22.1%)	147 (32.6%)	
High (>2000 USD)	22 (19.5%)	14 (3.1%)	
Preexisting physical disorders			
Hypertension	82 (72.6%)	254 (56.3%)	**0.002**
Dyslipidemia	65 (57.5%)	205 (45.5%)	**0.027**
Diabetes	41 (36.3%)	120 (26.6%)	**0.048**
Heart Diseases	48 (42.5%)	53 (11.8%)	**<0.001**
Atrial Fibrillation	34 (30.1%)	36 (8.0%)	**<0.001**
Asthma-COPD	44 (38.9%)	118 (26.2%)	**0.008**
Cancer	2 (1.8%)	15 (3.3%)	0.545

The variables that are not present in the table did not show a significant association with ischemic stroke. Numbers in bold indicate significant value of *p*s.

#### Bivariate analysis of other factors associated with ischemic stroke

**Figure 2:**
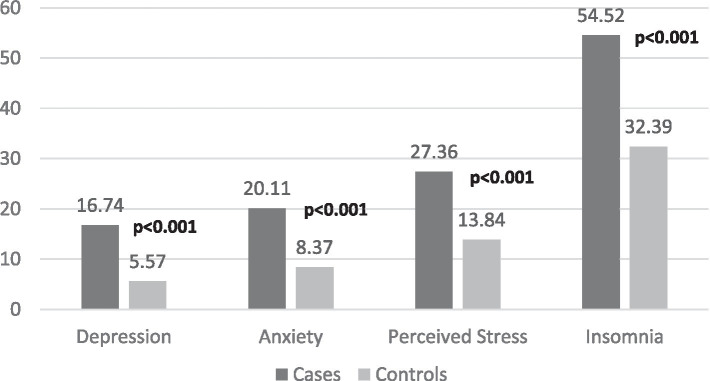
Comparison between cases and matched-controls in terms of mental health problems. Numbers in bold indicate significant value of *p*s.

Our findings suggest that ischemic stroke patients had significantly greater incidences of antecedent depression, anxiety, PS, and insomnia than controls (Figure 2).

Our results also showed that both ER strategies are strongly associated with the presence of ischemic stroke (Table 2).

**Table 2:** Bivariate analysis of participants’ tendency to regulate their emotions and ischemic stroke.

Variable	Presence of ischemic stroke (N = 113)	Absence of ischemic stroke (N = 451)	Value of *p*	Mean ± SD	
Cognitive reappraisal (CR)	17.97 ± 3.42	29.77 ± 8.44	**<0.001**
Expressive suppression (ES)	12.07 ± 2.32	19.88 ± 5.66	**<0.001**

Numbers in bold indicate significant *p* values.

#### Multivariable analysis: Logistic regression

All significant parameters from the bivariate analysis were included in the multivariable logistic regression. The model was suitable, and the Hosmer-Lemeshow test was adequate.

According to the outcomes of our regression model, depression (adjusted odds ratio [aOR]: 1.232, 95% confidence interval [CI]: 1.008–1.506), PS (aOR: 1.690, 95% CI: 1.413–2.022), low educational level (aOR: 0.335, 95% CI: 0.011–10.579) and being married (aOR: 3.862, 95% CI: 1.509–9.888) were all associated with an increased risk of suffering an ischemic stroke, whereas anxiety, insomnia, the CR and the ES approach was not identified as being implicated in the ischemic stroke risk in the final step model (Table 3).

**Table 3:** Adjusted odds ratios with their 95% confidence intervals from the logistic regression of ischemic stroke among cases and control.

Logistic regression taking the presence vs. absence of ischemic stroke as the dependent variable and taking depression, anxiety, PS and insomnia, cognitive reappraisal and expressive suppression as independent variables.			
Variables	*p*	aOR	95% CI
Depression (HDRS)	**0.041**	1.232	1.008–1.506
Anxiety (HAMA)	0.059	0.803	0.640–1.009
Perceived stress (PS)	**<0.001**	1.690	1.413–2.022
Insomnia (LIS)	0.076	1.037	0.996–1.079
Marital status (Yes vs. No)	**0.005**	3.862	1.509–9.888
Educational level (High vs. Low)	**0.004**	0.335	0.011–10.579

Backward Stepwise Likelihood ratio method; Logistic regression. Variables entered: Age, Marital status, Educational Level, Hypertension, Dyslipidemia, Diabetes, Heart Diseases, Atrial Fibrillation, Asthma-COPD, Depression, Anxiety, Perceived stress, Insomnia, Cognitive Reappraisal and Expressive suppression. aOR: Adjusted odds ratio; CI: 95% Confidence Interval; Numbers in bold indicate significant *p* values.

#### Moderation analysis

In this analysis, we investigated whether the choice of ER strategy may moderate the impact of the psychological disease and therefore influence the occurrence of an ischemic stroke. The moderated logistic regression incorporated all significant parameters from the bivariate analysis. The model was sufficient, as was the Hosmer-Lemeshow test.

The CR method was found to decrease the risk of ischemic stroke by acting as a significant positive moderator in the link between PS (Table 4: Model 3: CR*PSS: aOR: 0.978, 95% CI: 0.966–0.990), insomnia (Table 4: Model 4: CR*LIS: aOR: 0.994, 95% CI: 0.989–1.000; Model 5: CR*LIS: aOR: 0.986, 95% CI: 0.977–0.995) and ischemic stroke.

**Table 4:** Adjusted odds ratios with their 95% confidence intervals from the moderated logistic regression of ischemic stroke among cases and control.

Model 1: Moderated logistic regression taking the presence vs. absence of ischemic stroke as the dependent variable and taking depression, cognitive reappraisal, expressive suppression, CR*depression and ES*depression as independent variables.			
Variables	*p*	aOR	95% CI
ES*HDRS	**<0.001**	1.013	1.009–1.017
Cognitive reappraisal (CR)	**<0.001**	0.777	0.75–0.834
Marital status (Yes vs. No)	**<0.001**	4.801	2.437–9.457
Educational level (High vs. Low)	**0.001**	0.287	0.36–0.606
Model 2: Moderated logistic regression taking the presence vs. absence of ischemic stroke as the dependent variable and taking anxiety, cognitive reappraisal, expressive suppression, CR* anxiety and ES*anxiety as independent variables.			
Variables	*p*	aOR	95% CI
ES*HAMA	**<0.001**	1.015	1.010–1.021
Expressive suppression (ES)	**<0.001**	1.676	0.605–1.756
Marital status (Yes vs. No)	**<0.001**	4.408	2.284–8.506
Educational level (High vs. Low)	**<0.001**	0.234	0.113–0.488
Model 3: Moderated logistic regression taking the presence vs. absence of ischemic stroke as the dependent variable and taking perceived stress, cognitive reappraisal, expressive suppression, CR* PSS and ES*PSS as independent variables.			
Variables	*p*	aOR	95% CI
Perceived stress (PS)	**<0.001**	2.0981	2.105–4.222
CR*PSS	**<0.001**	0.978	0.966–0.990
Cognitive reappraisal (CR)	**<0.001**	0.652	0.259–2.167
Marital status (Yes vs. No)	**<0.001**	4.908	2.191–10.993
Educational level (High vs. Low)	**<0.001**	0.184	0.075–0.455
Model 4: Moderated logistic regression taking the presence vs. absence of ischemic stroke as the dependent variable and taking insomnia, cognitive reappraisal, expressive suppression, CR* insomnia and ES* insomnia as independent variables.			
Variables	*p*	aOR	95% CI
Insomnia (LIS)	0.062	1.099	0.995–1.214
CR*LIS	**0.050**	0.994	0.989–1.000
ES*LIS	0.102	1.009	0.998–1.019
Expressive suppression (ES)	0.061	0.725	0.518–1.015
Marital status (Yes vs. No)	**<0.001**	3.690	1.890–7.207
Educational level (High vs. Low)	**0.017**	0.388	0.178–0.844
Model 5: Moderated logistic regression taking the presence vs. absence of ischemic stroke as the dependent variable and all of the direct impact connected to the independent variable and the two moderators as independent variables.			
Variables	*p*	aOR	95% CI
Anxiety (HAMA)	0.082	0.908	0.815–1.012
Perceived stress (PS)	**<0.001**	3.232	2.205–4.736
Cognitive reappraisal (CR)	**0.001**	0.964	0.964–0.982
ES*PSS	**<0.001**	1.599	1.200–2.132
CR*LIS	**0.002**	0.986	0.977–0.995
ES*LIS	**0.001**	1.024	1.010–1.038
Marital status (Yes vs. No)	**<0.001**	4.397	1.951–9.909
Educational level (High vs. Low)	**0.001**	0.187	0.071–0.496

aOR: Adjusted odds ratio; CI: 95% Confidence Interval; Numbers in bold indicate significant *p* values.

The ES strategy, on the other hand, was found to be a significant negative moderator on the link between depression (Table 4: Model 1: ES*HDRS: aOR: 1.013, 95% CI: 1.009–1.017), anxiety (Table 4: Model 2: ES*HAMA: aOR: 1.015, 95% CI: 1.010–1.021), PS (Table 4: Model 5: ES*PSS: aOR: 1.599, 95% CI: 1.200–2.132), insomnia (Table 4: Model 5: ES*LIS: aOR: 1.024, 95% CI: 1.010–1.038) and ischemic stroke, resulting in an increased risk of stroke incidence.

### Effect of variables on stroke severity

#### Bivariate analysis of variables associated with the levels of stroke severity

A significantly higher percentage of married participants had a minor/moderate stroke, and those with a moderate to severe/severe stroke had a lower educational level. Preexisting physical disorders, such as hypertension, dyslipidemia, heart diseases, atrial fibrillation, asthma-COPD were substantially more common among moderate to severe/severe stroke patients. When compared to those with no stroke or minor/moderate stroke severity, people with moderate to severe/severe stroke had a higher mean age and a higher mean of all preexisting psychological disorders studied. Significant use of emotion regulation strategies was prevalent among stroke-free individuals. There was no significant relationship between stroke severity and the following variables: gender, diabetes, and cancer (Table 5).

**Table 5:** Bivariate analysis of variables associated with the levels of stroke severity.

Variable	Levels of stroke severity			*p* value
	No stroke	Minor /moderate stroke	Moderate to severe /severe stroke	
	Mean ± SD			**<0.001**
Age	62.88 ± 11.6	59.40 ± 10.7	75.00 ± 6.2	
Gender	N (%)			0.772
Male	203 (45.0%)	33 (47.8%)	18 (40.9%)	
Female	248 (55.0%)	36 (52.2%)	26 (59.1%)	
Marital status				**0.027**
Single/divorced /widowed	165 (36.6%)	14 (20.3%)	14 (31.8%)	
Married	286 (63.4%)	55 (79.7%)	30 (68.2%)	
Educational level				**<0.001**
Primary-complementary	141 (31.3%)	29 (42.0%)	32 (72.7%)	
Secondary	164 (36.4%)	9 (13.0%)	11 (25.0%)	
University	146 (32.4%)	31 (44.9%)	1 (2.3%)	
Preexisting physical disorders				
Hypertension	254 (56.3%)	49 (71.0%)	33 (75.0%)	**0.006**
Dyslipidemia	205 (45.5%)	36 (52.2%)	29 (65.9%)	**0.025**
Diabetes	120 (26.6%)	24 (34.8%)	17 (38.6%)	0.109
Heart Diseases	53 (11.8%)	19 (27.5%)	29 (65.9%)	**<0.001**
Atrial fibrillation	36 (8.0%)	16 (23.2%)	18 (40.9%)	**<0.001**
Asthma-COPD	118 (26.2%)	25 (36.2%)	19 (43.2%)	**0.019**
Cancer	15 (3.3%)	1 (1.4%)	1 (2.3%)	0.744
	Mean ± SD			
Preexisting psychological disorders				
Depression (HDRS)	5.57 ± 5.5	14.40 ± 5.0	20.41 ± 6.1	**<0.001**
Anxiety (HAMA)	8.37 ± 6.7	18.32 ± 4.7	22.9 ± 3.9	**<0.001**
Perceived stress (PS)	13.84 ± 8.1	26.84 ± 2.4	28.18 ± 1.7	**<0.001**
Insomnia (LIS)	32.38 ± 11.6	51.30 ± 9.3	59.57 ± 7.7	**<0.001**
Emotion regulation				**<0.001**
Cognitive reappraisal (CR)	29.77 ± 8.4	17.90 ± 3.6	18.09 ± 3.2	**<0.001**
Expressive suppression (ES)	19.88 ± 5.6	12.03 ± 2.5	12.14 ± 2.0	**<0.001**

Numbers in bold indicate significant *p* values.

#### Multivariable analysis: Multinomial regression

Our regression analysis revealed that being married versus single (aOR: 3.725, 95% CI: 1.468–9.455), having a low level of education versus a high level (aOR: 0.123, 95% CI: 0.35–0.436), having PS (aOR: 1.618, 95% CI: 1.319–1.986), and having heart diseases (aOR: 5.775, 95% CI: 1.435–23.251) were significantly associated with an increased likelihood of having a minor/moderate stroke compared to people who had never had a stroke (Table 6: Model 1).

**Table 6:** Multivariable analysis: Multinomial regression taking the levels of stroke severity.

Model 1: Levels of stroke severity (Minor /moderate stroke *vs* no stroke)			
Variables	*p*	aOR	95% CI
Age	0.947	0.943	0.897–0.991
Marital status (married vs. single*)	**0.006**	3.725	1.468–9.455
Educational level (secondary vs. primary-complementary*)	0.204	0.454	0.134–1.535
Educational level (university vs. primary-complementary*)	**0.001**	0.123	0.350–0.436
Depression (HDRS)	0.360	0.993	0.818–1.207
Anxiety (HAMA)	0.333	0.887	0.696–1.131
Perceived stress (PS)	**<0.001**	1.618	1.319–1.986
Insomnia (LIS)	0.113	1.039	0.991–1.089
Cognitive reappraisal (CR)	0.509	0.881	0.605–1.283
Expressive suppression (ES)	0.821	1.063	0.625–1.808
Hypertension (yes vs. no*)	0.106	2.074	0.857–5.020
Dyslipidemia (yes vs. no*)	0.764	1.149	0.464–2.844
Heart diseases (yes vs. no*)	**0.014**	5.775	1.435–23.251
Atrial fibrillation (yes vs. no*)	0.066	3.469	0.922–13.056
Asthma-COPD (yes vs. no*)	0.174	1.835	0.765–4.403
Model 2: Levels of stroke severity (Moderate to severe /severe stroke *vs* no stroke)			
Variables	*p*	aOR	95% CI
Age	**0.049**	1.103	1.000–1.216
Marital status (married vs. single*)	0.051	4.452	0.993–19.953
Educational level (secondary vs. primary-complementary*)	0.077	0.109	0.009–1.267
Educational level (university vs. primary-complementary*)	0.660	0.701	0.143–3.423
Depression (HDRS)	**0.021**	1.088	0.747–1.586
Anxiety (HAMA)	0.660	0.868	0640–1.176
Perceived stress (PS)	**<0.001**	2.564	1.604–4.100
Insomnia (LIS)	0.731	1.016	0.927–1.114
Cognitive reappraisal (CR)	0.908	1.031	0.614–1.731
Expressive suppression (ES)	0.506	0.773	0.362–1.651
Hypertension (yes vs. no*)	0.582	0.643	0.134–3.091
Dyslipidemia (yes vs. no*)	0.607	1.405	0.384–5.134
Heart diseases (yes vs. no*)	**0.011**	10.657	1.724–65.883
Atrial fibrillation (yes vs. no*)	0.105	3.560	0.767–16.531
Asthma-COPD (yes vs. no*)	0.080	2.998	0.875–10.266

*Reference group; aOR: Adjusted odds ratio; CI: 95% Confidence Interval; Numbers in bold indicate significant value of *p*s.

The odds of a moderate to severe/severe stroke were significantly higher with age (aOR: 1.103, 95% CI: 1.000–1.216), depression (aOR: 1.088, 95% CI: 0.747–1.586), PS (aOR: 2.564, 95% CI: 1.604–4.100), and heart diseases (aOR: 10.657, 95% CI: 1.724–65.883) compared to people who had never had a stroke (Table 6: Model 2).

## Discussion

In the current study examining the association between psychological disorders and stroke risk, the bivariate analysis revealed that the incidence of ischemic stroke was greater among patients with depression, anxiety, PS, and insomnia. Importantly, patients who had suffered from depression or PS at any time prior to the onset of stroke were found to have a significant impact on stroke risk. In addition, since ER was linked to an increased risk of ischemic stroke, a moderation analysis was conducted to determine whether it influences the independent variables studied, specifically depression, anxiety, PS, and insomnia. On the other hand, the severity of a stroke was found to be strongly correlated with a history of depression and PS, which was another significant finding from this research.

Many studies, including meta-analyses and systematic reviews, concluded that depression plays an important role in increasing the risk of stroke (67), regardless of other risk factors such as hypertension and diabetes, reinforcing the idea that the association between depression and stroke could be independent of coexisting vascular diseases (68). This result was similar to our findings in this study. Besides, a previous systematic review found no distinguishing clinical features between early and late-onset depression and ended up finding a balanced risk of stroke in both young and elderly individuals with depression (69). It is possible that increased stroke risk in depressed people is caused by pharmacological interferences with platelet aggregation caused by antidepressant medication (70). Furthermore, previous research has suggested that underlying cerebral pathomechanisms were specific to depression disorder, such as cerebral inflammation, dysregulation of the hypothalamic–pituitary–adrenal axis, increased platelet reactivity, and autonomic dysfunction (71). In addition, studies have shown that managing depressive symptoms may reduce the risk of stroke (72). As a result, the aforementioned mechanisms may be the prevailing cause of the increase in stroke risk observed in our study, and whether treating depressive symptoms could lower the likelihood of stroke warrants additional research (73).

This study’s findings on the effect of PS on stroke incidence were consistent with those of many other studies. The latter found that high levels of stress were associated with an increased risk of ischemic stroke (74–77), and this association varied depending on the type of stroke (36) and was greater in patients who already had vascular risk factors (37). Furthermore, a study found that the risk of stroke increased 15 days after psychiatric hospitalization (57). Stress, on the other hand, was discovered to be an overestimated risk factor for stroke due to a lack of clear tools for objective measurement and validation of the methods based on a similar study (77).

Two large studies, including the Rotterdam study, found a weak association between anxiety and the risk of ischemic stroke (18, 23). These findings were similar to ours, which revealed no significant relationship between anxiety disorders and stroke risk, despite the fact that anxiety symptoms were only related to stroke in a short-term interval in a study (23), asserting that further investigation is needed.

According to research, insomnia, sleeping patterns, and the risk of stroke are all highly correlated (26, 31, 78). Indeed, short and long sleep duration, as well as sleep-related movement disorders, have been shown to be potential risk factors for cardiovascular events and stroke, as well as a major cause of mortality (26), particularly in young adults (31). Moreover, sleep disturbances and disorders have seemed to be both a risk factor for stroke and to be worsened by a stroke (78). In our research, the unacquired insomnia-stroke relationship could be explained by the fact that the participants were mostly elderly, with a mean age of more than 62 years, and when looking at people suffering from insomnia, the risk of stroke was especially observed in young adults.

Albeit the largest number of studies implying that the reappraisal strategy is advantageous (79–81) there have been situations where the suppression strategy has been beneficial, particularly during short times and interpersonal relationships (82). Overall, experimental and individual differences research supported the critical role of CR and ES in both adaptive and dysfunctional emotional processing and regulation (83–85). Additionally, structural and functional brain studies showed a resulting brain network made up of target regions for various emotional regulation processes (86–90). According to the findings of these studies on the effects of ER strategies, CR was shown to have a healthier profile of short-term affective, cognitive, and social consequences than ES (40, 44, 46–48). Given that, the link between emotional regulation and stroke found in our study could be explained by the fact that the brain structural basis and functional activation were connected to the habitual use of CR and ES. Other studies, on the other hand, have found that habitual suppression of thoughts and emotions may contribute to depression and anxiety disorders, whereas reappraisal was associated with less depression, less negative affect, and increased life satisfaction (90–92). Moreover, promising theoretical and practical evidence for the early intervention of depressed or anxious individuals and preventing or relieving stress-related difficulties was discovered in these studies when evaluating the effects of emotional regulation strategies on anxiety, depression, or other pathologies by training patients in CR or even positive reappraisal (91–101). In light of these effects, a moderation study was conducted to investigate the potential modulating effect of these strategies.

Our moderation analysis, which included five different models, revealed that ES had a significant moderating effect on depression (model 1), anxiety (model 2), PS (model 5), and insomnia (model 5), resulting in an increased risk of stroke incidence. In contrast, CR decreased the risk of ischemic stroke by exerting a significant moderating effect on the following independent variables: PS (model 3), insomnia (models 4 and 5). These findings could be explained by the fact that ES, rather than CR, may play a crucial role in the experience of stress-related symptoms, as previously determined by research (94). Moreover, the strong relationship between PS and CR, as well as the positive impact on ischemic stroke risk, could be explained by the fact that reappraisal directly refers to the elaboration of consciousness content, such as feelings or interpretations. As a result, reappraisal is more efficient in changing subjective experience, especially since it is less cognitively costly than suppression (102). In addition, ER was found to be closely related to the insomnia-ischemic stroke risk relationship. Indeed, in a cross-sectional study, Steptoe et al. discovered that positive emotions predicted better sleep quality after controlling for health, socioeconomic status, and psychopathology (103). Moreover, Mauss et al. noticed a link between poor sleep and impaired ER ability. Poor sleep quality in the previous week, in particular, was linked to decreased CR in their study (104).

Besides, our multinomial regression model revealed that pre-stroke depression and PS were significant risk factors for stroke severity, thereby increasing an individual’s vulnerability to the disease. However, there is a paucity of studies examining the relationship between pre-stroke psychiatric morbidity and stroke severity or post-stroke outcome. Two studies have found correlations between pre-stroke depression, stroke severity, and functional outcome (50–52). Our findings are in line with these studies, providing evidence of an association between pre-stroke depressive symptoms and stroke severity. Regarding PS, there is a lack of attention paid to the potential role of PS in the development of stroke, with very few studies investigating its relationship with stroke severity or outcome after stroke. However, a mechanism for this has not yet been proposed, and other explanations may be at play, so it is difficult to draw firm conclusions from our findings. Possible mechanisms include PS’s influence on vascular inflammation, oxidative stress, or immune dysfunction; all three play a role in the pathophysiology of vascular disease (105). In fact, PS is related to increased catecholamine release and sympathetic activation, which may either directly or indirectly affect the vascular system (105). Additionally, PS adversely affects immune responses (106), which may result in increased susceptibility to stroke complications and thus may contribute to explaining the association between PS and severe stroke cases in particular. Taken together, there appear to be a variety of plausible mechanisms that may explain the association between PS and stroke severity.

## Limitations and strengths

The study presents several limitations. First, due to the case–control nature of our study, our observations should be interpreted with caution. As a result, recall bias may be present, particularly in patients with stroke, which may lead to giving false information when filling out the questionnaire since stroke may affect the patients’ recall memory and when seeking personal information from their guardian. Second, we lacked prescription data on psychiatric drugs such as antidepressants and anti-anxiety medications, which have been linked to an increased risk of stroke, as well as possible drug–drug interactions, which could have influenced our findings. Moreover, personal medical history was regarded as a potential risk factor for ischemic stroke in our study, but we did not look into the types of medical drugs used to treat these medical illnesses, which could also be a risk factor for stroke, as well as participant adherence to these drugs. Another eventual limitation of the study is that our data was gathered through self-report measures, which raises the chance of participant response bias, particularly post-stroke, where they could be tired and not vigilant enough when answering questions. In addition, when dealing with mental illnesses, patients and controls who were questioned may have been in denial or provided socially approved responses. Besides that, caregivers who were close to the stroke patient may have been unaware of their personal conditions or may have given socially desirable responses. Further, we were unable to construct the exact number of patient and proxy respondents in our study, and since proxy responses have small but statistically significant differences from patient responses, this could have increased response bias. Thus, future prospective research combining self-report and ecological momentary assessments may be more informative in clarifying the link between depression, anxiety, PS, insomnia, ER, and stroke risk.

Another significant downside is the likelihood of selection bias. Individuals who refused to participate, as in any volunteer study, may have characteristics similar to the general population; in terms of context, this generates a sample of controls that may not be representative of general population exposure. Additionally, when participants were queried about the reasons for their rejection or even partial responses, it was mostly due to the fact that psychiatric diseases are an unpleasant and highly confidential issue.

Our research, on the other hand, has several strengths. To begin with, we used standardized questionnaires to assess independent variables such as depression, anxiety, insomnia, PS, and ER, which reduced the possibility of missing patients’ comorbidities. In addition, by executing two regression models with distinct dependent variables, we were able to evaluate the impact of these variables on both stroke risk and severity. Furthermore, we were able to perform moderation analysis, which allowed us to determine whether the effect of the studied psychological diseases on ischemic stroke is consistent or varies across two types of ER. Although this is an observational study, it appears that people suffering from mental illnesses should pay more attention to their physical health and require ongoing monitoring from their physician.

## Clinical implications

Research is still needed to outline more in depth the pathophysiological mechanisms of stroke and their relationship with depression, anxiety, insomnia, and PS; thus, future studies must mainly implement a longitudinal cohort design with appropriate follow-up periods and rigorous checking of exposure and outcomes. Furthermore, whether our findings are related to the natural history of these diseases, medical treatments will need to be studied further in future researches. These studies should focus on classifying stroke patients according to the TOAST classification (107) and evaluating the impact of each TOAST subtype of stroke on the association between these diseases and ischemic stroke risk, preferably enlisting individuals who are free of cerebrovascular and cardiovascular diseases and providing longitudinal cerebral structural and functional assessment. Furthermore, more detailed measures are proposed to provide insight into which subcomponents of these illnesses appear to be significant and might even lead to a substantial association not observed in this study.

Further to that, future research should focus on both CR and ES, refining terminology and exploring the convergent and divergent properties within clinical samples. In addition, given the strong overlap between depression, anxiety, insomnia, and PS, the role of ER in the development and persistence of these illnesses-associated reactions and symptoms, particularly its relationship with the risk of ischemic stroke, warrants further investigation. As a result, we must consider whether ES is more than just a moderator in the relationship between the independent variable and stroke, for instance, a causal factor of stress-related symptoms, or rather a correlate that subsequently resulted from the presence of other factors or the utilization of other inept ER strategies.

Besides that, there may be clinical implications, as intervention strategies that can determine whether changes in one of these ways of regulating emotions may not lead to modifications in the other and whether they are truly independent. Given this, it is worth investigating whether increasing reappraisal could reduce the conceivably maladaptive use of expressive suppression.

Additionally, our study emphasizes the importance of healthcare providers being on the lookout for signs of potential depression, anxiety, PS, and insomnia in their patients, particularly those at high risk of stroke, and investigating whether prompt intervention can reduce the risk of ischemic stroke. Lastly, since the stroke severity was found to be strongly correlated with a history of depression and PS, this suggests that individuals with a history of depressive and PS symptoms are more likely to experience a severe stroke; therefore, it is important for clinicians to be aware of these potential risk factors and consider them when designing interventions to improve outcomes after stroke by implementing endeavors to screen at-risk patients for post-stroke aggravated symptoms, initiating treatment, and developing more effective strategies to promote rehabilitation participation and secondary prevention strategy adherence.

## Conclusion

In conclusion, depression and PS are considered to be significant risk factors for ischemic stroke among adult patients in the Lebanese population. Additional research is needed to determine whether early detection, prevention, and treatment of these modifiable risk factors can lead to a decreased risk of stroke. Since pre-stroke depression and PS were also found to be strongly correlated with stroke severity, future studies should evaluate the association between pre-stroke depression, PS, and stroke severity in order to gain a deeper understanding of the complex interaction between these variables. Furthermore, the present results indicate that ES use may promote depression, anxiety, and stress, increasing their association with stroke, whereas CR may play a protective role in stress and insomnia and their association with stroke. It remains to be seen whether this method can be used to support and reinforce their treatment strategies, as well as whether there is a true impact of this method on depression and anxiety, which were not found in our study.

In this context, given the influential role of these psychological diseases and ER strategies, practicing psychiatrists and other mental healthcare practitioners must be advised of and urged to be aware of the increased risk of stroke development in patients with these diseases and assess their ER with the aim of generating more integrated treatments. In addition, these conclusions can inform clinical decisions, such as the need to pay closer attention to the long-term outcome of stroke patients with depression and PS.

## Data availability statement

The raw data supporting the conclusions of this article will be made available by the authors, without undue reservation.

## Ethics statement

The studies involving human participants were reviewed and approved by Ethics committee of the Psychiatric Hospital of the Cross. The patients/participants provided their written informed consent to participate in this study.

## Author contributions

EM and SH designed the study, carried out the analysis, and interpreted the results. EM drafted the manuscript. PS and HH reviewed the paper. All authors reviewed the final manuscript, contributed to the article, and approved the submitted version.

## Conflict of interest

The authors declare that the research was conducted in the absence of any commercial or financial relationships that could be construed as a potential conflict of interest.

## Publisher’s note

All claims expressed in this article are solely those of the authors and do not necessarily represent those of their affiliated organizations, or those of the publisher, the editors and the reviewers. Any product that may be evaluated in this article, or claim that may be made by its manufacturer, is not guaranteed or endorsed by the publisher.

## Abbreviations

PS: perceived stress
ER: emotion regulation
CR: cognitive reappraisal
ES: expressive suppression
SPSS: statistical package for social science
HDRS: Hamilton depression rating scale
HAMA: Hamilton anxiety rating scale
PSS: perceived stress scale
LIS: Lebanese insomnia scale
ERQ: emotion regulation questionnaire
